# Effect of Nitrogen Dopant Agents in the Performance of Graphene-Based Cathodes for Li-S Batteries

**DOI:** 10.3390/nano14060489

**Published:** 2024-03-08

**Authors:** Adrián Licari, Almudena Benítez, Juan Luis Gómez-Cámer, Rafael Trócoli, Álvaro Caballero

**Affiliations:** Departamento de Química Inorgánica e Ingeniería Química, Instituto Químico para la Energía y el Medioambiente (IQUEMA), Facultad de Ciencias, Universidad de Córdoba, 14071 Córdoba, Spain; q82lipea@uco.es (A.L.); jl.gomez@uco.es (J.L.G.-C.); rafael.trocoli@uco.es (R.T.)

**Keywords:** graphene-based material, nitrogen functionalisation, ethylenediamine, ammonia, lithium-sulphur battery

## Abstract

Lithium-sulphur (Li-S) batteries offer high energy density compared to lithium-ion batteries, emerging as a promising technology for the next generation of energy storage systems. The ongoing challenge is to improve their electrochemical performance, extend their useful life and mitigate some problems that persist in this technology, by the investigation in materials with diverse properties. This work seeks to elucidate the importance and repercussions associated with functionalisation of graphene-based materials through nitrogen incorporation (more than 9 wt.% N), employing different chemical agents such as ethylenediamine and ammonia. Herein, differences in both the textural properties and the chemical environment of nitrogen within the carbonaceous network are identified, resulting in distinct electrochemical behaviours. The electrochemical performance of electrodes prepared from ammonia-functionalised samples surpasses that of ethylenediamine-functionalised samples in terms of both efficiency and rate performance. Conversely, the ethylenediamine-functionalised samples excel in stability, showing exceptional values in capacity retention per cycle. The outcomes exceeded expectations in energy performance, allowing the Li-S cells to be subjected to ultra-high rate cycling while maintaining appropriate capacity values.

## 1. Introduction

Currently, urgent measures are needed to reduce our dependence on fossil fuels. Despite the implementation and adoption of clean and renewable energy technologies over the years, such as solar, wind or hydro power, around 84% of the primary energy that is consumed still comes from natural gas, oil or coal [1]. This prevalence persists due to the intermittent nature of renewable energy sources, requiring energy storage systems to harness energy from solar and wind sources. This discrepancy arises from a misalignment between the peak energy consumption of society and the peak generation of these renewable energy sources. At present, the most popular energy storage system in the world is the lithium-ion (Li-ion) battery [2]. Many authors are still researching this technology, but Li-ion batteries are reaching their limit in terms of practical specific energy, and they are not good enough to meet the requirements for their booming applications [3,4]; therefore, other alternatives are being investigated. For this purpose, it is noteworthy to highlight the development of lithium-sulphur (Li-S) batteries. Although not yet widely available commercially, these batteries present several advantages, such as: (i) high specific theoretical capacity (1675 mAh g^−1^, which is equivalent to about 2600 Wh kg^−1^ of specific energy density) [5] and (ii) greater lightness, which is substantial when designing the battery pack for use in electric vehicles—a lower weight in the battery pack implies that a greater number of batteries can be added to the vehicle and, therefore, its capacity and autonomy increase [6]. Furthermore, sulphur is an abundant element in the Earth’s crust and is very low-cost because it is mainly generated as a by-product of the petrochemical industry. In addition, sulphur is a material with low toxicity and low environmental impact, unlike cobalt, nickel or manganese, elements currently used in the manufacturing of Li-ion batteries [5].

Despite the strengths of this technology, there are some weaknesses that must be overcome to reach a certain level of maturity. These challenges mainly focus on the low conductivity of sulphur and the lithium sulphide that is formed, the volume variation throughout the charge and discharge cycles, the formation of lithium dendrites on the negative electrode and the loss of active material in the cathode caused by the dissolution of long-chain lithium polysulfides at the electrodes, known as the shuttle effect [5,6,7,8]. Among the problems mentioned, the shuttle effect stands out; it causes the migration of the intermediate reaction products towards the negative electrode, transforming them into insoluble species in the electrolyte (Li_2_S_2_ and Li_2_S) that can remain deposited on the surface of the lithium metal, preventing them from returning to the cathode and, therefore, causing a progressive loss of active material. In addition, the deposition of these species on the lithium surface can lead to anode corrosion, thus increasing the internal resistance of the circuit [9]. To mitigate this effect, a wide variety of solutions have been tested, from modifying the separator [10], creating a solid electrolyte interphase (SEI) in the cathode that prevents the migration of Li polysulfides [9], adding different additives to the electrolyte [11] or incorporating conductive and porous matrices that can host the sulphur in the cathode, the last of which has become one of the best perspectives due to its successful results [12]. This can be achieved through the use of large surface area materials with micro- and mesoporosity where sulphur can infiltrate, allowing for the adsorption of lithium polysulfides [13].

As a proposal, one material that incorporates some of the previously mentioned characteristics is graphene. Graphene consists of a 2D material based on a single sheet of carbon atoms with a hexagonal configuration. Some of its electrical properties are high conductivity of the charge carrier, high thermal conductivity and an unusual quantum Hall effect [14,15]. The combination of graphene’s properties has boosted its application in different technological sectors [16,17,18,19,20]. In addition, the surface of graphene can be functionalised with different kinds of heteroatoms to modify the electrical, optical, chemical and physical properties of graphene. In the literature, other authors have described the use of these graphene-based materials functionalised with different heteroatoms (N, O, S, P, B, Cl and F, or combinations of them) for Li-S batteries to upgrade the overall functionality of the battery [21,22,23,24,25,26].

Nitrogen-functionalised graphene, which has been extensively studied in the energy storage field, has proven to be a very efficient way to improve the electrochemical performance of Li-S cells. Qiu et al. reported a high surface area nitrogen-doped graphene that exhibited commendable capacities at high rates and demonstrated low capacity loss during cycling, but employed an exceptionally low sulphur loading on the electrodes (0.8 mg cm^−2^) [27]. Song et al. described the synthesis of nitrogen-doped graphene sheets utilising cyanamide as a nitrogen source, which was mixed with graphene oxide (GO) and thermally reduced at 600 °C under Ar and H_2_ conditions. This method achieved an ultra-high pore volume (5.4 cm^3^ g^−1^); however, it is an expensive technique and the percentage of nitrogen present in the sheets was not highlighted (6.53 wt.% N) [28]. Jia et al. successfully synthesised a nitrogen-doped graphene aerogel with a notably higher nitrogen content (9.23 wt.% N) using a simplified methodology compared to that employed by other researchers. Nevertheless, the electrochemical characteristics of nitrogen-functionalised graphene have not yet been studied adequately and in-depth in Li-S batteries, with galvanostatic measurements being limited to only 50 or 100 cycles in most studies [29]. This fact is evident in the study by J.-H. Kang et al. [30], where a graphene functionalised with ethylenediamine was cycled at 0.1C for only 80 cycles. In contrast, other researchers have surpassed the 100-cycle threshold, exemplified by the work of L. Li et al. [31], who examined graphene functionalised with ammonia at 0.5C in Li-S cells, achieving notable capacity values of 700 mAh g^−1^ after 300 cycles. Both ethylenediamine and ammonia offer specific advantages depending on the goals of functionalisation and the desired final properties of graphene [30,31].

This work presents for the first time a comparative analysis employing ethylenediamine and ammonia as functionalising agents, which have demonstrated the ability to incorporate a substantial content of nitrogen into the graphene matrix (~9.5 wt.% N). The synthesis of nitrogen-functionalised graphene-based materials has been achieved through a simplified and cost-effective method, distinguishing it from methodologies previously used by other researchers. Additionally, sulphur inclusion was accomplished via a melt-diffusion process, resulting in composites with a significant sulphur percentage (70 wt.% S). Subsequently, an extensive electrochemical study was conducted using electrodes with moderate sulphur content (2.5 mg cm^−2^). This research aims to enhance comprehension of the benefits associated with utilising graphene-based materials functionalised with nitrogen. These materials are postulated as highly promising candidates for advancing the development of Li-S batteries, thereby expanding the prospects of their commercialisation in the near future.

## 2. Materials and Methods

### 2.1. Synthesis of Graphene Oxide (GO)

Graphene oxide (GO) was synthesised by the modified Hummers method [32]. To do this, 3 g of commercial graphite (Sigma-Aldrich, St. Louis, MO, USA), 70 mL of H_2_SO_4_ (Panreac, Barcelona, Spain) and 1.5 g of NaNO_3_ (Sigma-Aldrich) were mixed and stirred for 20 min. Afterwards, 9 g of KMnO_4_ (Sigma-Aldrich) were added, preventing the temperature from exceeding 20 °C. The temperature was then increased to 35 ± 5 °C and stirred for 30 min. After, 140 mL of distilled water was added while stirring continuously and maintaining a temperature of 90 ± 5 °C for 30 min. Then, 500 mL of distilled water and 15 mL of a 3% H_2_O_2_ solution (Sigma-Aldrich) were added. Once the solution was tempered, it was brought into contact with 250 mL of 10% HCl (Panreac, 37%) and then centrifuged and washed with distilled water repeatedly until a neutral pH (≥5.8) was reached. The obtained gel was dried in an oven at 60 °C for 12 h. Finally, the solid was ground in a planetary ball mill (Retsch PM 100, Haan, Germany) at 500 rpm for 3 h with rotation inversion every 15 min to homogenise and pulverise the GO sample.

### 2.2. Synthesis of Nitrogen-Functionalised rGO via Ethylenediamine (en-rGO)

The functionalisation of reduced graphitic oxide via ethylenediamine (en-rGO) consisted of two steps: impregnation and hydrothermal reduction with modifications in accordance with the process previously reported in the literature [33]. The impregnation consisted of preparing a suspension of GO (15 mg mL^−1^) dispersed in 10 mL of an aqueous solution of ethylenediamine (en), maintaining a weight ratio of 2:1 (GO:en; *w*:*w*). To promote the dispersion of this mixture, it was sonicated for 30 min in an ultrasonic bath. Then, the dispersion was stirred for 2 h and poured into a Petri dish, which was placed in an oven at 70 °C for 4 h. The second step consisted of a hydrothermal reduction in which the pretreated GO (2 mg mL^−1^) was dispersed in an ethylenediamine solution (10 mg mL^−1^) with a total volume of 45 mL of ethanol. This dispersion was then placed in a 90 mL capacity autoclave reactor and heated at 180 °C for 12 h. Finally, the contents extracted from the autoclave were filtered, washed with water and dried at 80 °C [33,34].

### 2.3. Synthesis of Nitrogen-Functionalised rGO via NH_3_ (NH_3_-rGO)

The synthesis method used to obtain ammonia-functionalised reduced graphene oxide (NH_3_-rGO) was based on a hydrothermal treatment with adaptations in line with findings documented in the prior literature [35]. Specifically, a colloidal suspension of GO (2 mg mL^−1^) was dispersed in an aqueous solution of ammonia (1:1; *v*:*v*) using ultrasonication, and then the autoclave reactor was placed in an oven at 180 °C for 12 h. Subsequently, it was cooled, filtered and washed with distilled water. Finally, the powdered sample was dried at 80 °C [35,36].

### 2.4. Synthesis of Reduced Graphene Oxide (rGO)

With the purpose of evaluating the functionalisation of the previously prepared rGO against a reference material, reduced graphene oxide (rGO) was synthesised by performing the hydrothermal reduction of GO, but without the incorporation of any nitrogen source. Specifically, a colloidal suspension of 2 mg mL^−1^ of GO in ethanol was prepared by ultrasonication and subsequently placed in a 90 mL capacity autoclave reactor. The reactor was heated in an oven at 180 °C for 12 h. Once the autoclave was tempered, the contents were filtered under vacuum and washed with plenty of distilled water. Finally, the obtained rGO powder was dried in an oven at 80 °C [33].

### 2.5. Synthesis of the Graphene-Based Materials and Sulphur Composites

The previous graphene-based samples were mixed with sulphur using the melt-diffusion method to obtain the composite samples. To do this, the carbon and sulphur precursors were predried in a vacuum oven (Buchi, Flawil, Switzerland) at 120 and 45 °C, respectively, to completely remove moisture. The samples were then placed in an inert atmosphere glove box (MBraun 150, München, Germany). Once inside, the selected graphene-based material was homogeneously blended with sulphur in a weight ratio of 30:70 (rGO:S), a fitting proportion often considered suitable for studies in Li-S batteries. The resulting mixture was then transferred into an autoclave. Subsequently, the autoclave was heated in an oven at 155 °C for 24 h. Finally, the composite samples were ground manually until the particle size was homogenised. The composites were denoted as S@en-rGO, S@NH_3_-rGO and S@rGO [37].

### 2.6. Cathode Electrode Preparation and Cell Assembly

An electrode mixture, or slurry, was prepared by combining different components: the graphene–sulphur composite, Super P carbon (or carbon black, Timcal, Bodiio, Switzerland) as a conductive additive and polyvinylidene fluoride (PVDF, Gelon, Shan Dong, China) as a binder, in a weight ratio of 80:10:10, respectively. Then, the mix was ground for homogenisation and the necessary amount of N-methyl-2-pyrrolidone (NMP, Acros Organics, Geel, Belgium) was added to achieve the desired consistency. Next, the slurry was deposited on a conductive substrate using the tape casting technique. The electrode was then dried at 45 °C overnight to evaporate the dispersing agent. Afterwards, it was cut into 12.8 mm diameter discs, which were thoroughly dried in a vacuum oven (Buchi) at 45 °C for 3 h, removing any trace of moisture before being placed in an inert atmosphere glove box (Inert IL-4GB, Amesbury, MA, USA) for cell assembly. The specific sulphur loading on the electrode was approximately 2.5 mg cm^−2^ [38].

**Scheme 1:**
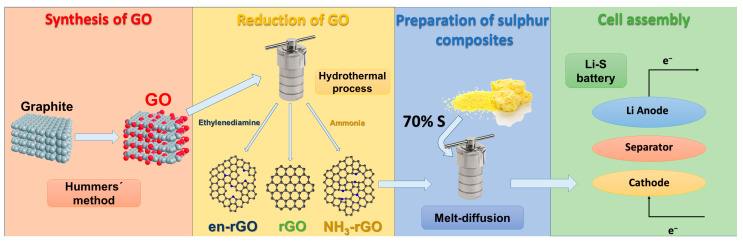
N-doped graphene-based materials as cathode for Li-S batteries.

Once the cathode was prepared, the Li-S cells were assembled into coin-type devices (standard commercial model CR2032). The cells were manufactured by facing the cathode material under study against lithium metal (Gelon) as an anode; finally, a polyethylene separator membrane (Celgard 2400, Charlotte, NC, USA) was placed between the two electrodes in which the electrolyte was impregnated. The electrolyte formulation consisted of the dissolution of two lithium salts, lithium bis(trifluoromethanesulfonyl) imide (LiTFSI 1 M, Sigma Aldrich) and lithium nitrate (LiNO_3_ 0.4 M, Panreac), in a mixture of the organic solvents 1,2-dimethoxyethane (DME, Sigma Aldrich) and 1,3-dioxolane (DOL, Sigma Aldrich) (1:1; *v*:*v*). The volume of electrolyte added per mg of sulphur was 25 μL. Once all the elements were arranged inside the cell, the Li-S cell was hermetically sealed using a hydraulic press. Scheme 1 summarises the work diagram regarding the preparation of materials and electrodes.

### 2.7. Characterisation and Electrochemical Measurements

X-ray diffraction (XRD) analysis was performed using a Bruker D8 Discover diffractometer (Bruker, Berlin, Germany) with monochromatic Cu-Kα radiation. The diffractograms were recorded by scanning between 5 and 80° (2*θ*), using a step size and time of 0.040° and 1.05 s per step, respectively. Raman spectra were recorded on a Renishaw inVia (Renishaw, London, UK) spectrometer equipped with a Leica microscope (Leica, Wetzlar, Germany), a Renishaw CCD Camera detector (578 × 400) and a 532 nm laser. Raman spectra were carried out in the range between 200 and 2000 cm^−1^. The laser intensity was 1%, the exposure time was 10 s and 10 scans were performed per sample. Nitrogen adsorption–desorption isotherms were carried out at liquid nitrogen temperature (−196 °C) using a Micromeritics ASAP 2020 (Micromeritics, Norcross, GA, USA). The degassing of GO was carried out at 50 °C for 24 h, while the rGO samples were heated at 150 °C for 6 h. The methods used for the calculation of specific surface area and pore size distribution were Brunauer–Emmett–Teller (BET) theory and density functional theory (DFT), respectively. The morphology of synthesised materials was observed by scanning electron microscopy (SEM) and transmission electron microscopy (TEM). TEM images were recorded on a JEOL JEM-1400 (JEOL Ltd., Akishima, Japan), while SEM images were made with a JEOL JSM-7800F with an X-ACT detector for EDX elemental microanalysis. Post-mortem SEM images were taken from electrodes of cells cycled at increasing rates (C/10, C/8, C/5, C/3, C/2, 1C and 2C), which had been previously disassembled in a glovebox. The equipment used to perform the X-ray photoelectron spectroscopy was a Phoibos 150 MCD XPS (SPECS group, Berlin, Germany) spectrometer with a monochromatic Al source. CasaXPS software (version 2.3.26) was used to process the spectra. For the CN elemental analysis, the instrument used was a Eurovector EA 3000 (Eurovector, Pavia, Italy elemental analyser with automatic injector. Thermogravimetric analysis (TGA) was carried out with a TGA/DSC-1 analyser (Mettler Toledo, Greifensee, Switzerland). Regarding the conditions used for the carbonaceous samples, the heating ramp was 10 °C min^−1^ in a temperature range between 30 °C and 900 °C, using an oxygen or nitrogen atmosphere (depending on the objective of the analysis) and a gas flow rate of 100 mL min^−1^ in both cases. However, TGA of rGO–sulphur mixtures was carried out only under a nitrogen atmosphere (flow rate of 100 mL min^−1^) with a temperature range between 30 °C and 600 °C and using the same heating ramp (10 °C min^−1^).

Galvanostatic measurements were performed on a 32-channel Arbin BT2143 potentiostat-galvanostat (ARBIN Instruments, College Station, TX, USA). A voltage window of 1.7–2.7 V vs. Li^+^/Li was set for these experiments. The current densities used in the extended cycling experiments were C/10 and C/5, with precycling at C/10 for the first 5 cycles (defining 1C as the current density needed to reach the theoretical capacity in 1 h; 1C = 1675 mA g^−1^). Galvanostatic cycling experiments were also programmed under increasing current densities (rate capability test), in which the following rates were used: C/10, C/8, C/5, C/3, C/2, 1C and 2C, finally returning to C/10. The sample functionalised by ammonia was exposed to a higher stress test in the electrochemical study, performing extended cycling chronopotentiometry at 1C and a rate capability test at C/10, C/5, C/2, 1C, 2C, 5C, 10C, 12C, 15C and returning to C/10. All rates and specific capacity values are with reference to the mass of sulphur in the electrode. Cyclic voltammetry (CV) and electrochemical impedance (EIS) measurements were performed on an Autolab PGSTAT-204 (Metrohm Autolab BV, Utrecht, The Netherlands). CV curves were recorded using different sweep rates (0.05, 0.1, 0.2, 0.4 and 0.6 mV s^−1^) in a potential window from 1.5 to 3.0 V. Impedance spectra were measured in open circuit voltage (OCV) and after the completion of 7 CV cycles, applying an amplitude of 10 mV within a frequency range between 500 kHz and 0.1 Hz. The measurements were processed by ZView 2.0 software.

## 3. Results and Discussion

### 3.1. Characterisation of Graphene-Based Samples

**Figure 1:**
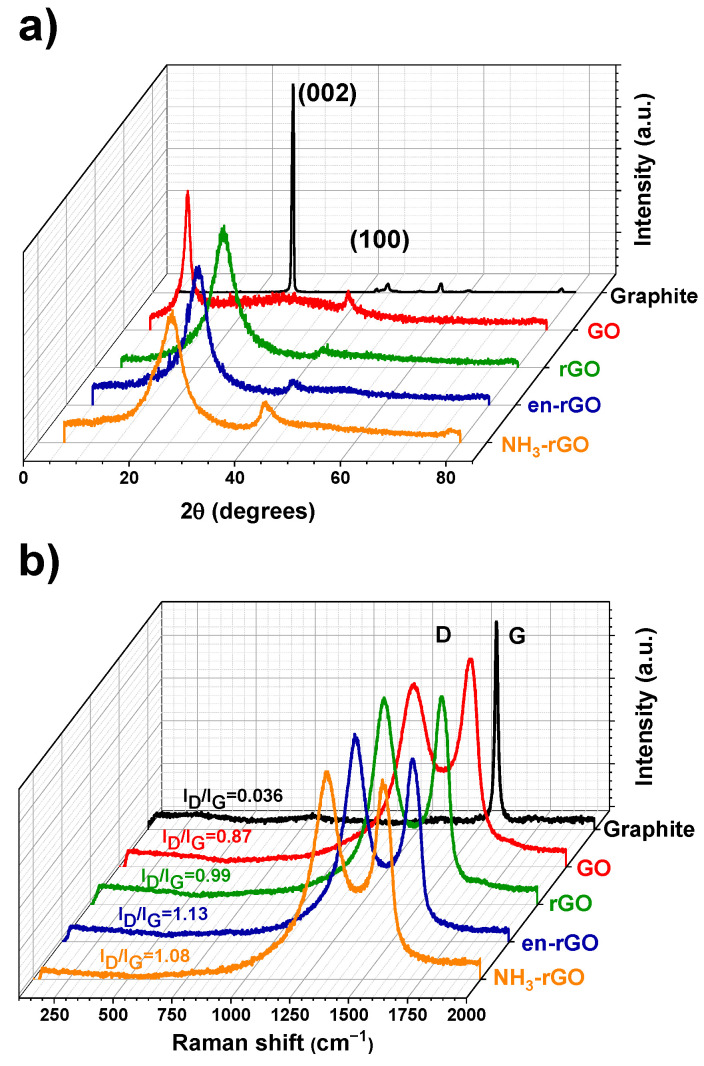
(**a**) X-ray diffraction pattern and (**b**) Raman spectra of several samples, including pristine graphite, GO, rGO, en-rGO and NH_3_-rGO.

A crucial stage in the production of graphene-based materials from graphitic oxide (GO) involves ensuring the complete reduction of this material. Therefore, the assessment of structural measurements becomes essential for evaluating the proper preparation of the samples. The X-ray diffraction (XRD) pattern of the GO, rGO, en-rGO and NH_3_-rGO samples and the pristine graphite, from which the synthesis of the other intermediates and final products starts, are shown in Figure 1a. The XRD pattern for commercial graphite provided two characteristic peaks of the graphite phase (JCPDS-PDF 12-0212) at ~26° and 41° (2*θ*), assigned to the 002 and 100 crystallographic planes, respectively [39]. After oxidation to GO, the reflection peak located at 26° (2*θ*) was shifted to a smaller angle, specifically at 11° (2*θ*), verifying an increase in the interlaminar spacing due to the insertion of functional groups and, therefore, confirming the correct exfoliation. Moreover, this peak was shifted again to ~26° (2*θ*) in the reduced samples, recovering the original position of the 002 plane in graphite, but with a remarkable broadening of the peaks that indicates a loss of crystallinity in the graphene sheets [40]. Raman spectra (Figure 1b) showed two characteristic bands at 1560 and 1360 cm^−1^, corresponding to G and D bands, respectively. The G band stands for the stretching vibration E2g mode, while the D band originates from defects within the structure of the graphite [41]; therefore, the ratio between the intensity of both bands (I_D_/I_G_) determines the degree of disorder. I_D_/I_G_ ratios for the graphite, GO, rGO, en-rGO and NH_3_-rGO samples were 0.036, 0.87, 0.99, 1.13 and 1.08, respectively, which confirmed a high ordering in the lamellar structure of commercial graphite, a slight increase in disorder in the GO sample and a deeper increase in disorder in the reduced samples [42]. These values are notably high when compared to other carbons, particularly those derived from biomass [43,44].

**Figure 2:**
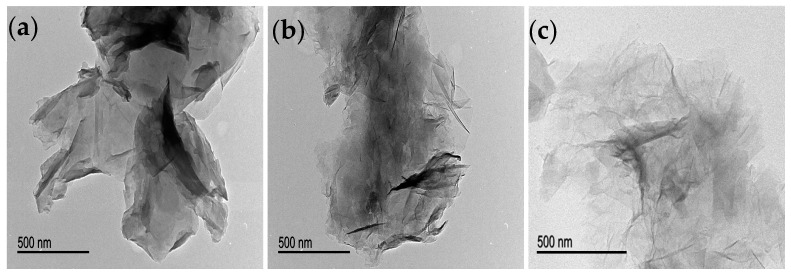
TEM images of (**a**) GO, (**b**) en-rGO and (**c**) NH_3_-rGO samples.

Verifying the acquisition of graphene-based materials also requires a comprehensive assessment of their morphology. Hence, an analysis of the samples was conducted using transmission electron microscopy (TEM, Figure 2). Specifically, Figure 2a illustrates an image of the GO sample, revealing a stack of smooth laminates. During the reduction process (Figure 2b,c), the GO sheets underwent exfoliation, resulting in wrinkled folds and three-dimensional configuration for graphene-based samples. Consequently, the morphology of both reduced samples showed similar morphological properties [42].

**Figure 3:**
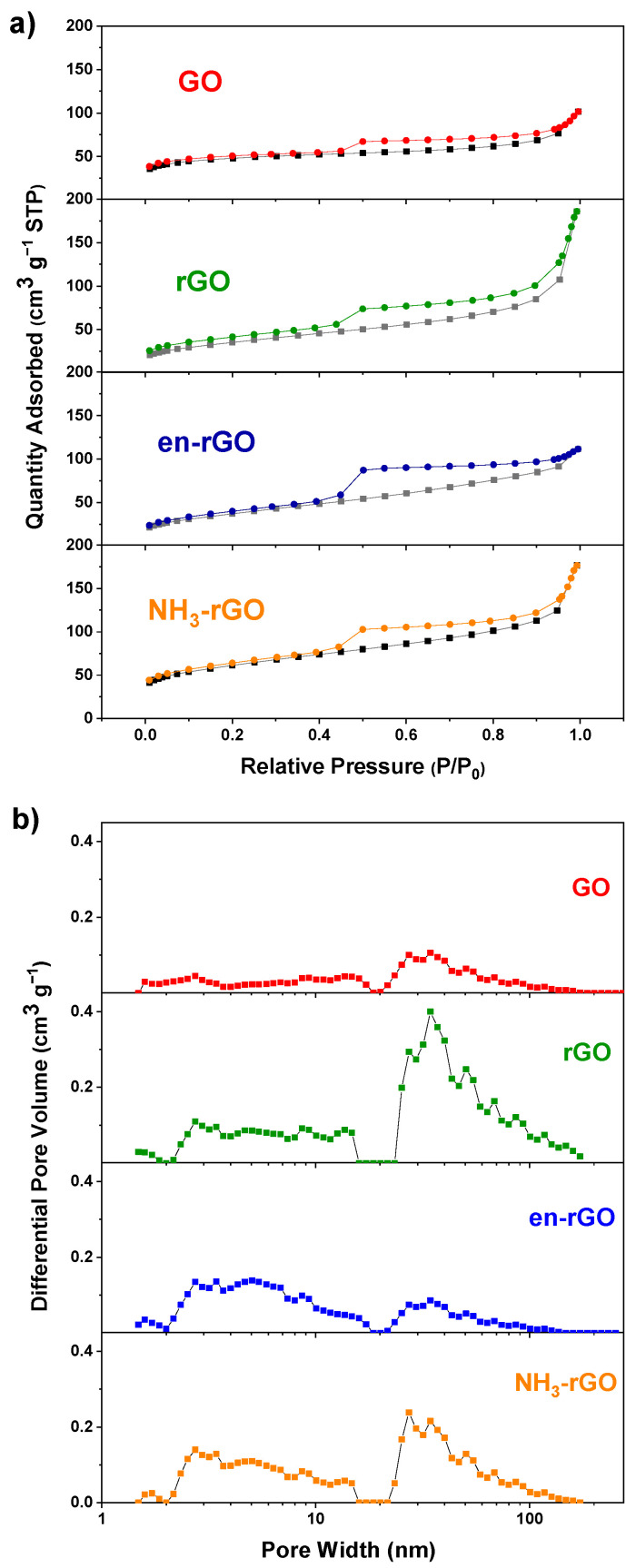
(**a**) Nitrogen adsorption/desorption isotherms and (**b**) pore size distribution of the GO, rGO, en-rGO and NH_3_-rGO samples.

Nitrogen adsorption/desorption isotherms were performed to evaluate the textural properties of the samples, and are represented in Figure 3a. All samples showed isotherm profiles conforming to type IV isotherms with hysteresis loop, according to BDDT classification [45], associated with mesoporous materials. In addition, Figure 3b illustrates the pore size distribution determined through the DFT (density functional theory) model, verifying the mesoporous nature of the samples. Notably, in both the rGO and NH_3_-rGO samples, a distinct region of macropores emerged, significantly influencing key surface properties such as total pore volume (V_T_) and surface area (S_BET_), as detailed in Table 1. Compatible with the previous literature, the remaining samples displayed S_BET_ and V_T_ values within the expected range for this sample type [46].

**Figure 4:**
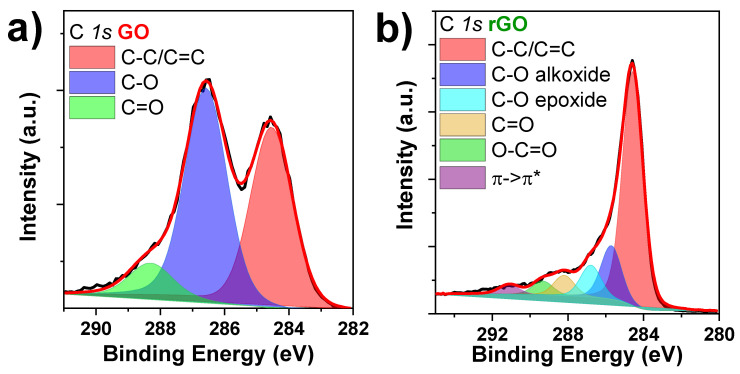

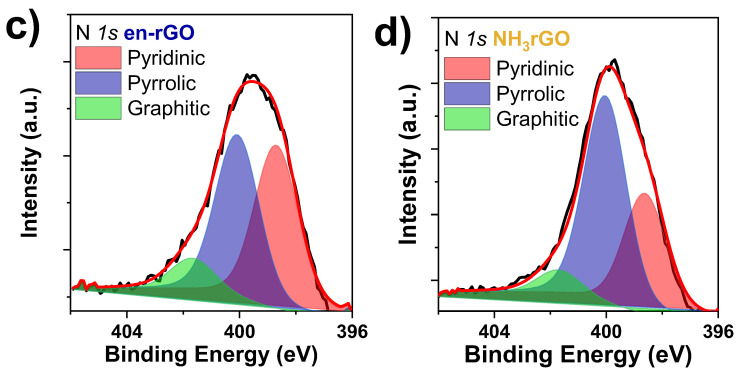
XPS spectra of the C*1s* signal for the (**a**) GO and (**b**) rGO samples, and the N*1s* signal for the (**c**) en-rGO and (**d**) NH_3_-rGO samples.

X-ray photoelectron spectroscopy (XPS) measurements provide information on the chemical environment and composition of the samples. In Figure S1a, the XPS survey of the different samples reveals the presence of C*1s* and O*1s* signals across all samples. Furthermore, in the case of the nitrogen-functionalised samples (en-rGO and NH_3_-rGO), an additional signal appears due to the contribution of N*1s*. In the C*1s* region of the GO sample (Figure 4a), there is a noticeably lower intensity in the C-C/C=C component compared to the rGO sample (Figure 4b), accounting for a contribution range of 42.5% to 64.4%. In contrast, more pronounced C-O interactions resulted for the GO sample due to the oxidation process and the presence of functional groups between the sheets of the GO structure. However, for the rGO sample, the C-O and C=O interactions decrease notably, accompanied by the emergence of π → π* interactions and a marked increase in the C-C interaction. These observations confirm an effective reduction of GO during the synthesis process of the samples. Additionally, C*1s* spectra are represented for en-rGO and NH_3_-rGO samples in Figure S1b,c, displaying a similar chemical environment to the rGO sample [47]. Moreover, Figure 4c,d depict the N1s spectra for the en-rGO and NH_3_-rGO samples, respectively, identifying three types of well-differentiated chemical environments corresponding to pyridinic, pyrrolic and graphitic nitrogen discernible at 398.6, 400.1 and 401.8 eV, respectively [48]. The proportions of these environments vary depending on the specific functionalising chemical agent. In the case of the sample prepared using ethylenediamine (en-rGO), the analysis reveals values of 9.02% for graphitic nitrogen, 45.49% for N-pyrrolic and 45.49% for N-pyridinic. Conversely, the sample synthesised with ammonia (NH_3_-rGO) exhibited different proportions: 6.82% for graphitic nitrogen, 58.96% for N-pyrrolic and only 34.22% for N-pyridinic. These results demonstrate a higher content of pyridinic nitrogen in the en-rGO sample, while the NH_3_-rGO sample exhibits a higher proportion of pyrrolic nitrogen [49]. As a complementary technique, a CN elemental analysis was performed on the reduced samples to determine the amount of carbon and nitrogen. In detail, the nitrogen content in rGO, en-rGO and NH_3_-rGO samples was 0.95, 9.21 and 9.41%, respectively.

**Figure 5:**
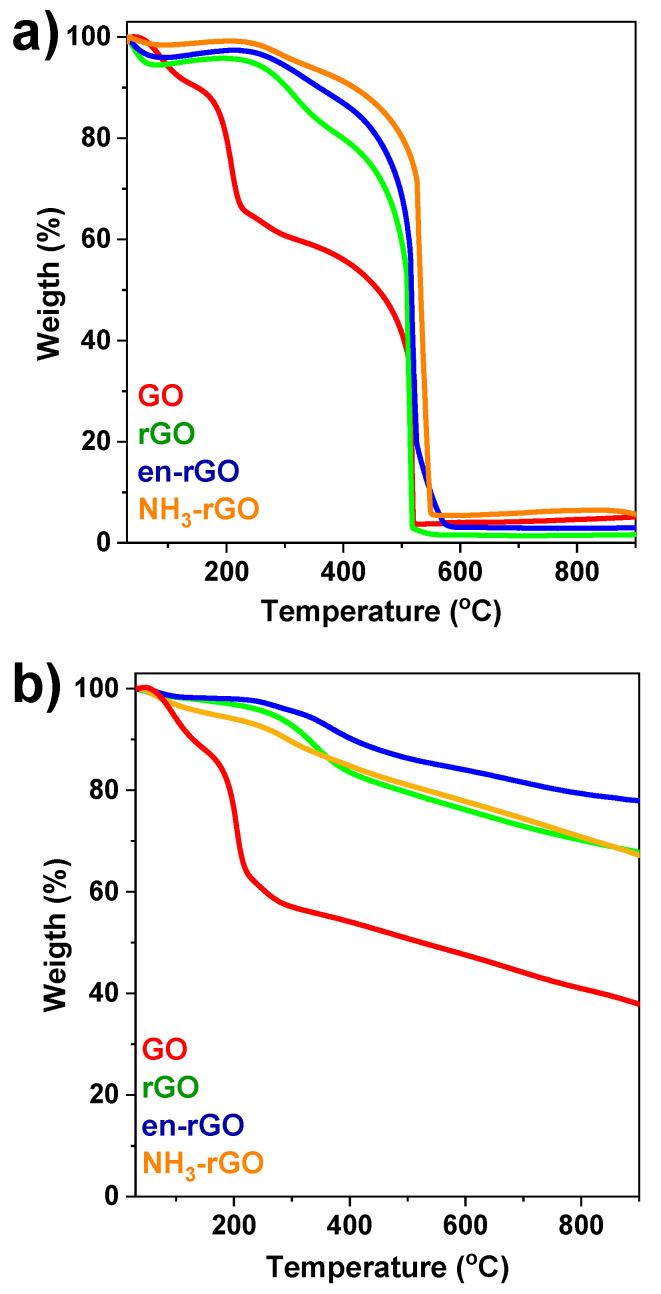
Thermogravimetric analysis of the GO, rGO, en-rGO and NH_3_-rGO samples in (**a**) oxygen and (**b**) nitrogen atmospheres.

Additionally, to gain deeper chemical insights into the thermal behaviour and composition of the samples, thermogravimetric analysis (TGA) was performed. In the case of measurements carried out under an oxygen atmosphere (Figure 5a), a pronounced decline in weight was observed above 350 °C for the rGO, en-rGO and NH_3_-rGO samples, aligning with carbon contents of 78.5%, 76% and 75%, respectively. Notably, the mineral matter content of all samples showed values below 5 wt.% and is attributed to impurities present in the commercial graphite used as the starting material. Additionally, a minor weight loss, occurring between 250 °C and 350 °C for the reduced samples (rGO, en-rGO and NH_3_-rGO) was attributed to the gradual loss of residual functional groups that still persisted in the structure, accounting for approximately 15%, 17% and 14% of the total weight, respectively. This percentage was notably lower compared to the GO sample, where functional group loss happened between 100 °C and 300 °C, with a content of around 35% [50]. The thermograms presented in Figure 5b under a nitrogen atmosphere exhibited a characteristic pattern for this type of sample, confirming comparable percentages of functional groups related with the weight loss observed between 200 and 400 °C. This alignment reinforces the finding from XPS analysis, highlighting the presence of a minimal proportion of residual functional groups in the graphene-based materials.

### 3.2. Characterisation of Sulphur Composites

**Figure 6:**
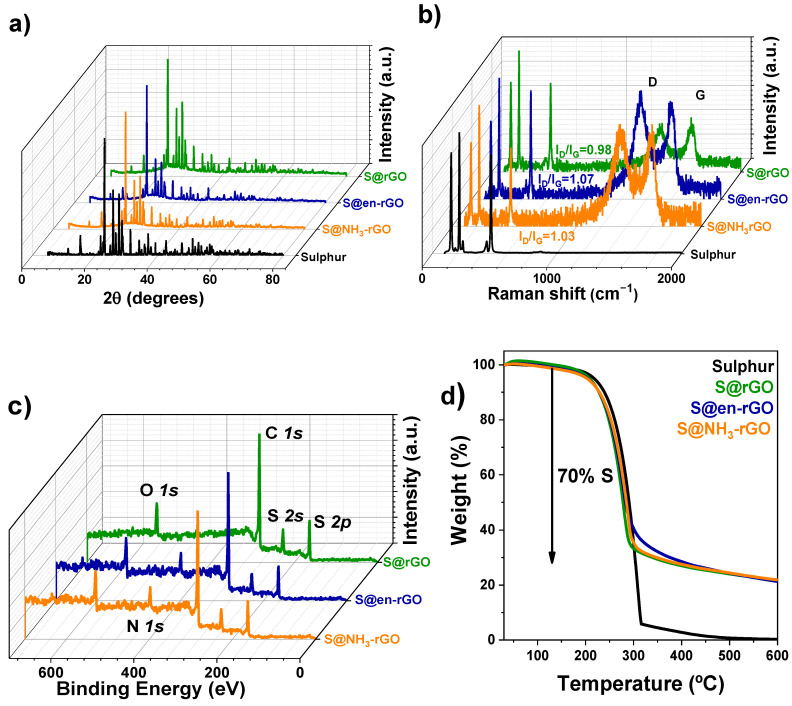
(**a**) XRD diffractogram, (**b**) Raman spectra, (**c**) XPS survey and (**d**) TGA of the S@rGO, S@en-rGO and S@NH_3_-rGO composites.

Following the analysis of the graphene-based samples, sulphur was introduced via the melt-diffusion method to form the sulphur composites (S@rGO, S@en-rGO and S@NH_3_-rGO). Various characterisation techniques, including XRD, Raman, XPS and TGA were employed to confirm sulphur’s presence within the structure, understand its chemical environment and determine the percentage of sulphur integrated into the pores of the graphene matrix (Figure 6). The evaluation of structural properties by XRD (Figure 6a) revealed multiple reflections assigned to the crystalline phase of orthorhombic sulphur (JCPDS-PDF 85-0799). These signals appeared intense and narrow, indicating a highly crystalline structure. Furthermore, a slightly broad band around 26° (2*θ*) corresponding to the disordered carbon of the graphene material used as a matrix is also appreciable. However, the Raman spectra (Figure 6b) prominently exhibited the D and G bands typical of disordered carbonaceous samples. In addition, Raman measurements confirmed the presence of elemental sulphur through three high-intensity signals below 500 cm^−1^, corresponding to characteristic orthorhombic S peaks (150, 219 and 474 cm^−1^) [51]. In the XPS spectra (Figure 6c), the signals related to the appearance of sulphur demonstrated its presence, and in Figure S2, the signal S*2p* has been highlighted to study the environment of the sulphur in the sample, where three signals appeared. Firstly, there was a doublet corresponding to S*2p_3/2_* and S*2p_1/2_* with an energy separation of 1.2 eV, indicating possible presence of C-S species. There was also a signal that could be ascribed to sulphates species [52]. EDX analysis, shown in Figure S3, was also carried out on the samples, indicating the existence of nitrogen in the composites based on the functionalised graphene-based samples. A homogenous dispersion of the sulphur in the composites, which is explained by the use of the melt-diffusion method, was also responsible for the formation of little particles of sulphur that facilitate the electrochemical performance of the electrodes [37]. Finally, once the presence of sulphur in the composite samples was verified, the amount of fixed sulphur in the graphene matrix was determined accurately using TGA. Figure 6c shows a weight loss stage occurring approximately between 200 °C and 400 °C, attributed to the sublimation of sulphur. This analysis revealed a consistent 70% by weight of elemental sulphur within the S@rGO, S@en-rGO and S@NH_3_-rGO composites. These results agree with the proportion of sulphur utilised during the preparation of these composite materials.

### 3.3. Electrochemical Study

**Figure 7:**
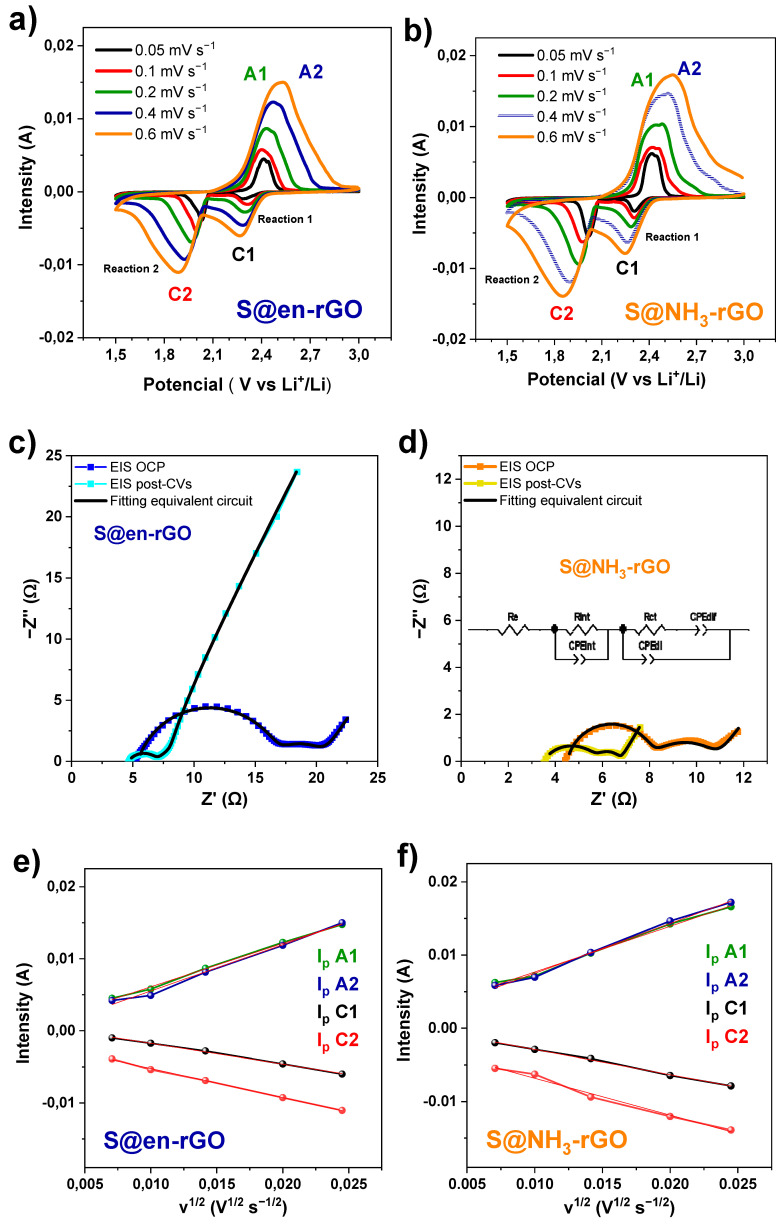
Cyclic voltammograms at 0.05, 0.1, 0.2, 0.4 and 0.6 mV s^−1^ between 3.0 and 1.5 V for (**a**) S@en-rGO and (**b**) S@NH_3_-rGO electrodes; impedance spectra of (**c**) S@en-rGO and (**d**) S@NH_3_-rGO electrodes, and the proposed equivalent circuit; graphical representation of peak intensity versus sweep speed elevated to a half for (**e**) S@en-rGO and (**f**) S@NH_3_-rGO electrodes.

Upon completion of the characterisation of the sulphur composites, they were employed in the fabrication of the cathode electrode for Li-S cells. Subsequently, an exhaustive electrochemical investigation was conducted on the different graphene-based materials functionalised with nitrogen. Figure 7a,b shows the cyclic voltammetry results of the N-functionalised samples recorded at several scan rates (0.05, 0.1, 0.2, 0.4 and 0.6 mV s^−1^) to examinate the reversibility of the electrochemical reactions and the stable working window. The discharge process is initiated, giving rise to two broad cathodic signals in each of the samples at 2.3 and 2.0 V. The first signal indicates the first state change (solid to liquid) and is attributed to the scission of the elemental sulphur ring (S_8_) with the subsequent formation of long-chain Li polysulfides, following the reaction: 2Li^+^ + (n)S+ 2e^−^ → Li_2_S_(n)_ (reaction 1) [8]. The second signal is associated with the second physical state change (from liquid to solid) during the electrochemical process, which corresponds to the transformation of the long-chain Li polysulfides into Li sulphide, giving rise to the following reaction: 2(n − 1)Li^+^ + Li_2_S_n_ + 2(n−1)e^−^ → nLi_2_S (reaction 2) [53]. As for the charging process, two anodic signals are observed at 2.41 and 2.44 V, which correspond to the same reactions indicated previously, but in the reverse direction. Thus, it is proved that the system is reversible for the three sulphur composites based on the different reduced samples (Figure S4a). Electrochemical impedance spectroscopy (EIS) was also performed on the Li-S cells; two measurements per sample were recorded, the first one at open circuit potential (OCP), and a second one after performing the CV cycles. Nyquist plots of the impedance spectra are shown in Figure 7c,d. All the measurements present two semicircles, one at high-frequency (HF) and the other at medium-frequency (MF), followed by a diffusion tail with the shape of a semi-infinite Warburg element. The proposed equivalent circuit for the totality of the samples is illustrated in Figure 7d, and the value of the resistance components of each measurement are collected in Table S1. In accordance with findings documented in the literature by other authors, the semicircle observed at HF can be attributed to the resistance generated at the interface of particles inside the electrode, representing internal resistance. This phenomenon is coupled with its associated capacitance and reproduces the electron conduction process from the current collector to the point where the charge transfer occurs [54]. If measurements of the same samples (OCP and post-CVs) are compared, it can be observed how the resistance value of the semicircle decreases once the cycle has been completed, as other authors have previously reported [40,55]. This fact can be justified based on two key phenomena. Firstly, in the OCP measurements, sulphur particles characterised by low conductivity are sandwiched between the particles of the graphene materials, which exhibit high conductivity. As cycling progresses, sulphur hosted within the electrode tends to be deposited closer to the interface with the electrolyte, particularly towards the end of the charging phase. Consequently, there’s an increase in interparticle contact within the electrode, primarily involving the graphene particles [54]. In addition, the sulphur now deposited on the cathode is already activated, implying it consists not only of orthorhombic sulphur, but also contains lithium polysulfide with higher conductivity. The latter has not been completely transformed into elemental sulphur, which favours electronic conduction between the particles and reduces the overall resistance of the process. The second semicircle described at MF is attributed to the charge transfer phenomenon, as previously reported [54]. In this case, a shorter time constant associated with these components (τ = RC; where *R* is the component of resistance of the circuit and *C* is the capacitance component of the circuit) of charge transfer signifies an easier occurrence of charge transfer. The values resulting from the calculation of τ, gathered in Table S1, consistently indicate a faster charge transfer on the S@NH_3_-rGO electrode, suggesting a potentially superior electrochemical performance. Finally, the circuit contains the constant phase element (CPE) associated with the diffusion of lithium ions in the electrolyte. It can be classified depending on its shape and, in this case, the diffusion component in all cases is closer to a semi-infinite Warburg type [56].

The diffusion coefficient of the lithium ions has been calculated in different ways. The first one considers the intensity of each signal and the cycling rate applied in the voltammogram using the Randles–Secvik method, which establishes that the adjustment of the maximum intensity of each signal is proportional to the square root of the speed (Equation (1)).

(1) Ip=2.69·105·n32·A·DLi12·CLi·v12

where I_p_ is the maximum intensity of each peak, n the number of electrons involved in each process, *A* the electrode area, D_Li_ the diffusion coefficient of the lithium ions, C_Li_ the concentration of lithium ions in the electrolyte and *v* the velocity applied in each of the voltammetry experiments. The linear fits for each of the samples are shown graphically in Figure 7e,f, and the diffusion coefficients calculated for each of the signals from the three electrode samples analysed are shown in Table S2. The diffusion coefficients are in ranges of values previously described by other authors for electrodes of a similar nature in Li-S batteries [57]. Comparatively, the electrode based on the sample functionalised with ammonia (S@NH_3_-rGO) stands out especially, presenting higher values of the diffusion coefficient. This fact may be related to the electrical and morphological properties of the material, and especially to its differentiated textural properties. The high specific surface area value of this graphene material will improve the contact between the electroactive material and the electrolyte of the cell, facilitating the diffusion of lithium ions during the charging and discharging processes of the battery. In addition, an alternative way to calculate the diffusion of lithium ions through this diffusion tail has been reported in the literature [58,59]; depending on the shape of the tail, the methodology changes. All the samples present a semi-infinite shape; therefore, the equation to find the diffusion coefficient will be the following (Equation (2)):

(2) $$D_{EIS}=\frac{1}{2}{\left[\frac{RT}{z^{2}F^{2}CA\sigma }\right]}^{2}$$

where D_EIS_ is the diffusion coefficient calculated by EIS, R is defined as the ideal gas constant (8.324 J mol^−1^ K^−1^), T is the temperature at which the measurement was taken (298.15 K), *z* is the number of electrons involved in the process, F is the Faraday constant (96,485 C mol^−1^), A is the area of the electrode, C is the concentration of lithium ions in the electrolyte and *σ* is the slope resulting from the linear fit (Figure S4d) of the real resistance versus the square root of the inverse of the frequency (Ω s^−1/2^) [60]. The diffusion coefficient values obtained from the Randles–Secvik calculation are presented in Table S2. Remarkably, these values align with those reported by other researchers who have utilised the same method for electrodes in Li-S cells [38]. Once again, the highest diffusion coefficient value corresponds to the ammonia-functionalised sample.

**Figure 8:**
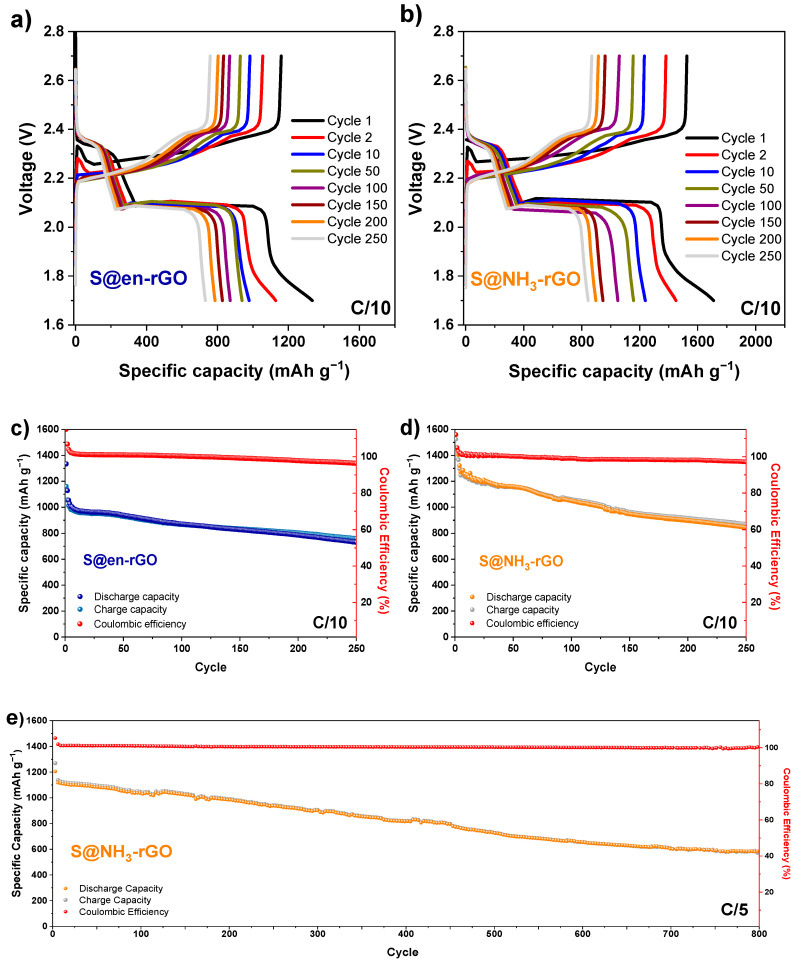
Charge/discharge profiles at a C/10 rate for 1, 2, 10, 50, 100, 150, 200 and 250 cycles with (**a**) S@en-rGO and (**b**) S@NH_3_-rGO electrodes. Long-term discharge/charge capacity values as a function of the cycle number and the Coulombic efficiency for (**c**) S@en-rGO and (**d**) S@NH_3_-rGO electrodes at a C/10 rate, and for (**e**) S@NH_3_-rGO electrode at a C/5 rate.

In order to complete the electrochemical study, galvanostatic charge/discharge measurements of the cells at different rates were carried out. Figure 8a,b shows the galvanostatic charge/discharge profiles of the Li-S cells for different samples at a cycling rate of C/10 (167.5 mA g^−1^). The discharge profiles present two different pseudo-plateaus, around 2.35 and 2.1 V, corresponding to the reduction signals described in the cyclic voltammetry curves. The charging curve also presents two pseudo-plateaus located at 2.2 and 2.35 V, which would correspond to the reactions in the reverse direction to those occurring in the discharge process. The shape of the profiles is maintained in the successive cycles, indicative of the reversibility of the electrochemical process. In addition, the variation in the values of the specific discharge capacity in cells with the functionalised samples (S@en-rGO and S@NH_3_-rGO) as a function of the number of cycles at a C/10 rate are shown in Figure 8c,d, respectively. It is evident that the specific capacity decreases progressively throughout cycling, with a more pronounced drop during the first cycles until reaching a certain level of stabilisation. The released capacity in the first cycle varies depending on the specific graphene electrode under study. Specifically, the electrode based on the S@NH_3_-rGO composite (Figure 8d) presents the highest specific capacity in the first cycle, with a value of 1708 mAh g^−1^. As cycling progresses, the specific capacity value decreases, and finally stabilises at an average capacity value of 1200 mAh g^−1^. On the other hand, the S@en-rGO composite (Figure 8c) presents a lower initial capacity electrode (1334 mAh g^−1^) and a lower stabilisation (~950 mAh g^−1^) than the S@NH_3_-rGO, suggesting that the content of pyrrolic nitrogen could have an electrocatalytic effect on the polysulfide’s reaction. Nevertheless, with an increasing number of cycles, the S@NH_3_-rGO electrode experiences a more pronounced decline in capacitance, exhibiting a capacity fade of 0.14% per cycle. The observed capacity fade in this context is likely attributed to the repeated cycling of the S@NH_3_-rGO electrode, causing a more significant decline in capacitance over time. This phenomenon can be associated with factors such as the shuttle effect, polysulfide dissolution, electrode instability or mechanical stresses during cycling, all of which contribute to the gradual loss of active material and, consequently, a decline in capacity [5,6,7,8]. In contrast, the S@en-rGO electrode exhibits a relatively lower capacity fade of 0.11% per cycle, suggesting that the chosen functionalisation approach may have mitigated some of the underlying causes of capacity deterioration.

However, as the number of cycles increases, the S@NH_3_-rGO electrode suffers a greater capacitance drop, with capacity fading by 0.14% per cycle; meanwhile, the S@en-rGO electrode suffers a lower capacity fading of 0.11% per cycle. From these data, it can be deduced that functionalisation with pyridinic nitrogen allows for higher specific capacity to be retained during prolonged cycling of the battery due to its superior ability to adsorb lithium polysulfides with greater strength [61].

**Figure 9:**
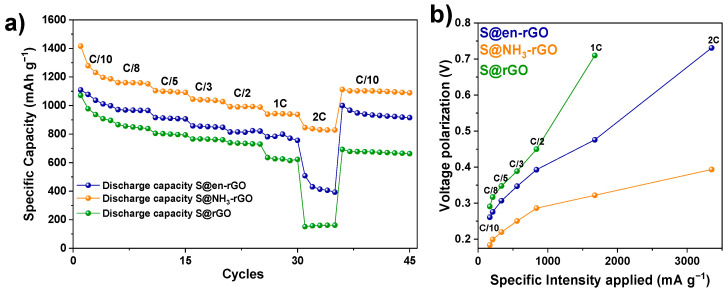
(**a**) Specific discharge capacity values as a function of the cycle number at C/10, C/8, C/5, C/3, C/2, 1C and 2C rates. (**b**) Values of the voltage polarisation as a function of the specific intensity applied for S@en-rGO, S@NH_3_-rGO and S@rGO electrodes.

This test was replicated at a higher rate, concretely, at C/5 (335 mA g^−1^), achieving, once again, an outstanding electrochemical performance for the S@NH_3_-rGO based electrode over 800 cycles, as illustrated in Figure 8e. It commenced with an initial specific capacity of 1867 mAh g^−1^, stabilising at around 1100 mAh g^−1^. Notably, this outperforms the cell employing the S@en-rGO electrode (Figure S5b), which could be attributed to the electrocatalytic effect of the pyridinic nitrogen on the polysulfides reaction, as was previously described [62]. The capacity fading behaviour remained consistent at C/5. Specifically, the S@NH_3_-rGO electrode exhibited a capacity fading of 0.077% per cycle; meanwhile, the S@en-rGO electrode showed only a 0.02% per cycle loss in specific capacity during the initial 80 cycles. Hence, this strengthens the idea that the S@en-rGO electrode effectively traps lithium polysulfides better than the S@NH_3_-rGO electrode. The higher specific capacity of the latter is attributed to the electrocatalytic effect, a phenomenon also evident in the rate capability test (Figure 9a). The capacity values exhibited by the electrode based on S@NH_3_-rGO surpass those of all other materials examined across all tested cycling rates. Notably, this electrode maintains consistent high-capacity values even at high cycling rates such as 2C (3350 mA g^−1^). These data confirm the excellent properties of the ammonia-functionalised graphene material as a sulphur matrix in Li-S technology. According to the conductivity and diffusion data, and as previously reported in the literature [62], this specific type of functionalised graphene material serves as a crucial factor in optimising the battery’s performance. It functions adeptly as an efficient matrix and an excellent electrocatalyst during the conversion reaction of lithium polysulfides. Notably, this capability extends to facilitating the transformation of lithium polysulfides even under demanding high cycling rates. The S@en-rGO electrode exhibits a gradual decrease in capacitance corresponding to an escalating current density, maintaining a consistent capacity decline. However, under the 2C rate, it suffers a pronounced capacity drop, although retaining a capacity of 400 mAh g^−1^. In contrast, the unfunctionalised electrode (S@rGO) undergoes a pronounced capacity decrease at 1C and becomes practically inefficient at 2C. Upon returning to the initial rate, remarkable capacity recovery is observed for both functionalised electrodes (S@en-rGO and S@NH_3_-rGO), achieving recovery values of 93.2% and 93.5%, respectively. In stark contrast, the unfunctionalised electrode (S@rGO) demonstrates a significantly lower recovery value, below 75%.

Furthermore, from the galvanostatic charge and discharge measurements, the voltage difference between the two processes can also be calculated by determining the cell polarisation values. Greater polarisation results in an increased overpotential, subjecting the cell to higher energy consumption and reduced energy density. This overpotential is increased by low electronic conductivity, low diffusivity of the lithium ions and high resistance to charge transfer [63]. As depicted in Figure 9b, the cell presenting the S@NH_3_-rGO electrode exhibits the lowest polarisation, confirming the results and conclusions derived from the CV, EIS and galvanostatic studies. These results highlight the superior conductivity, diffusivity and general electrochemical performance of the S@NH_3_-rGO electrode.

**Figure 10:**
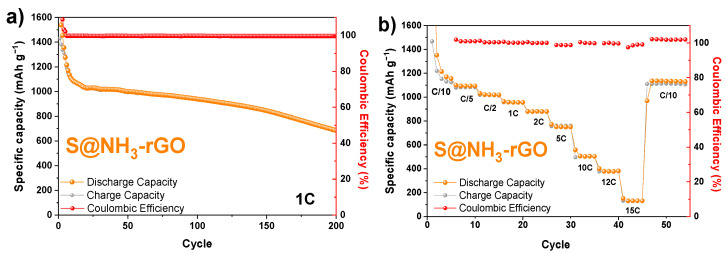
(**a**) Long-term cycling and Coulombic efficiency at a 1C rate over 200 cycles. (**b**) Values of the specific discharge capacity and Coulombic efficiency as a function of the number of cycles at C/10, C/5, C/2, 1C, 2C, 5C, 10C, 12C and 15C rates for the S@NH_3_-rGO electrode.

Owing to the excellent electrochemical performance delivered by the S@NH_3_-rGO composite, these electrodes were subjected to higher current densities in fast charging mode. First, galvanostatic charge/discharge measurements of these cells were completed at a cycling rate of 1C (1675 mA g^−1^) for 200 cycles, providing an initial capacity of 1760 mAh g^−1^. Figure 10a shows the variation of the specific charge and discharge capacity values as a function of the number of cycles, as well as the Coulombic efficiency. As cycling progresses, the specific capacity value gradually diminishes, eventually stabilising around 1000 mAh g^−1^, an outstanding performance value for such a high rate. This reinforces the hypothesis that the functionalisation of nitrogen via ammonia has an electrocatalytic effect on the polysulfide reaction at the cathode. So, in order to prove the limits of this electrocatalyst, some electrodes of S@NH_3_-rGO composite were prepared and assembled to be tested at an ultra-high rate (from C/10 to 15C rates). In Figure 10b, the outstanding electrochemical behaviour of the cell is evident up to 5C, achieving almost 800 mAh g^−1^. Beyond this point, the yield diminishes, but the cell continues to operate up to 12C, and the cell stops working at 15C, as illustrated in the charge and discharge profiles (Figure S6). It is worth highlighting the remarkable performance of the cell at ultra-high rates, such as 5C, where charge/discharge processes are completed in just 12 min.

The scanning electron microscopy (SEM) images of the S@NH_3_-rGO electrode, shown in Figure S7, represent the appearance of the electrode before and after cycling. Specifically, this electrode was recovered from a Li-S cell subjected to a rate capability test similar to that shown in Figure 9a (C/10, C/8, C/5, C/3, C/2, 1C and 2C rates at 5 cycles each; C/10 again during 50 more cycles). It is worth highlighting that minimal alteration in the morphology was observed after intense cycling. The electrode appeared more compact than before cycling, probably due to the pressure applied during cell assembly. Despite volume changes after undergoing various redox processes, no pulverisation of the particles was observed, even after multiple rate changes during the rate capability test. The absence of significant morphological alterations in the electrodes after postmortem analysis underlines their robustness and stability, positively contributing to their durability and long-term performance [57,64].

The results obtained from this electrochemical study demonstrate that the ammonia-functionalised material has shown remarkable performance due to the functionalisation with pyrrolic nitrogen and its electrocatalytic effect. The achieved results merit comparison with current state-of-the-art in Li-S batteries (highlighted in Table 2). As previously indicated, Qiu et al. [27] reported praiseworthy capacities at high rates and demonstrated low capacity loss during cycling, but an excessively low sulphur loading of 0.8 mg cm^−2^; meanwhile, the cells with graphene-based samples in this article have achieved similar results with higher sulphur loading (~2.5 mg cm^−2^), this being very necessary for a transition towards the industrialisation process of this technology. Song et al. [28] also showed a method of incorporating nitrogen into a graphenic matrix, but it proved to be a costly technique, and the percentage of nitrogen present was not emphasised (only 6.53 wt.% N). In this article, a successful functionalisation of graphene-based materials with nitrogen was achieved using different functionalisation agents (ammonia and ethylenediamine) and was capable of reaching a notably higher nitrogen content (9–9.5% wt.% N). Jia et al. [29] managed to obtain a nitrogen-doped graphene aerogel with a notably higher nitrogen portion using a simpler methodology, but the electrochemical characteristics of the reported material fell short of being satisfactory in Li-S batteries, since the galvanostatic measurements were conducted for only 50 or 100 cycles. In this article, the electrochemical characteristics were thoroughly studied, and galvanostatic measurements were tested at various current rates, including prolonged cycling (even up to 800 cycles), elucidating the true behaviour of these materials and taking a significant step towards the maturity of Li-S technology. While the functionalisation of graphene materials with ammonia and ethylenediamine has been documented in prior literature [30,31], the electrochemical findings from this study on the S@NH_3_-rGO and S@en-rGO material exhibited notable improvements over those reported in both referenced papers. The graphene-based material functionalised with ethylenediamine reported in the literature [30] only achieved 80 cycles at 0.1C, whereas in this study, 250 cycles are achieved at the same rate, with a decrease in capacity fading, as can be seen in Table 2. For the graphene-based material functionalised with ammonia, the study from the literature [31] only works for 100 cycles at 0.2C, while in this work, an ultra-long-term was conducted, reaching 800 cycles. Importantly, advancements are observed in key aspects such as specific capacity, number of cycles and rate reached, as can be seen in Table 2.

To conclude, the results have managed to provide new information about these materials through a comparative study involving various functionalisation agents. These findings pave the way for future cutting-edge research, with the use of this material as an astonishing sulphur host in Li-S batteries.

## 4. Conclusions

A comparative study between two nitrogen-functionalised graphene-based materials was carried out using ammonia and ethylenediamine as dopant agents. A complete characterisation of the materials and their sulphur composites was performed to evaluate their structural, textural, morphological and chemical properties. The synthesis method has been effectively validated, showing the success of both the Hummers method and subsequent hydrothermal reductions. This methodology demonstrates its ability to incorporate functional groups into the carbonaceous matrix, as well as the feasibility of employing ethylenediamine and ammonia as effective functionalising agents to introduce nitrogen as a heteroelement into reduced graphitic oxide within different chemical environments of the carbonaceous network. Sulphur composites were effectively prepared using a highly efficient method based on the diffusion of sulphur into the pores of the graphene matrix, achieving highly dispersed sulphur on the surface of the graphene-based materials with 70% sulphur content. Depending on the functionalising agent used, a different impact on the electrochemical performance was observed. Comprehensive electrochemical analysis showed that the ammonia-functionalised sample achieved superior electrochemical performance in terms of efficiency and rate performance, reaching average specific capacities at C/10 and C/5 rates of 1140 and 1080 mAh g^−1^, respectively. The remarkable performance is primarily attributed to nitrogen functionalisation within a chemically favourable environment, enhancing the electrocatalysis of lithium polysulfides during cycling. Additionally, it has superior textural properties for more efficient and effective accommodation of sulphur for electrochemical utilisation. On the other hand, the ethylenediamine-functionalised sample excels in terms of stability, achieving the most outstanding values in terms of capacity retention per cycle.

Therefore, ammonia-functionalisation of graphene introduces two relevant improvements in the performance of rGO-based cathodes for Li-S batteries: (i) an enriched presence of pyrrolic nitrogen induces substantial efficiency improvements, serving as an electrocatalyst for polysulfide conversion reactions; and (ii) a higher proportion of pyridinic-type nitrogen facilitates optimised adsorption of these lithium polysulfides, resulting in reduced capacity loss per cycle and enhanced battery stability. 

## Figures and Tables

**Table 1:** Surface area (S_BET_) and total pore volume (V_T_) values for GO, rGO, en-rGO and NH_3_-rGO samples.

Samples	S_BET_ (m^2^ g^−1^)	V_T_ (cm^3^ g^−1^)
GO	160	0.16
rGO	128	0.28
en-rGO	136	0.17
NH_3_-rGO	214	0.27

**Table 2:** A comparative analysis of the literature findings by other researchers on Li-S batteries with N-doped graphene-based materials as cathode.

N Content (wt.%)	S Content (wt.%)	S Loading (mg cm^−2^)	C-Rate	Cycle Number	Ci (mAh g^−1^)	Cf (mAh g^−1^)	Capacity Fading (% Cycle^−1^)	Ref.
3.9	60	0.8	0.2C	150	1200	750	0.25	[27]
6.53	80	~1.5	0.46C	300	1150	850	0.09	[28]
9.23	75	ND	0.1C	50	1200	880	0.53	[29]
ND	77	1.2	0.1C	75	1250	1050	0.21	[30]
7.1	70	2	0.2C	100	1200	850	0.29	[31]
9.21	70	2.5	0.1C	250	1000	740	0.11	This work
9.41	70	2.5	0.2C	800	1200	590	0.06	

Ci: initial capacity, after preliminary stabilization; Cf: final capacity; ND: not defined; capacity fading: decay rate per cycle.

## Data Availability

The data presented in this study are available on request to the corresponding authors.

## Supplementary Materials

The following supporting information can be downloaded at: https://www.mdpi.com/article/10.3390/nano14060489/s1, Figure S1. (a) XPS survey for the GO, rGO, en-rGO and NH_3_-rGO samples; XPS spectrum of the C*1s* signal for the (b) en-GO, and (c) NH_3_-rGO samples. Figure S2. XPS spectrum of the *S2p* signal for the (a) S@en-GO, (b) S@NH_3_-rGO, and (c) S@rGO samples. Figure S3. SEM images of the (a) S@rGO, (d) S@en-GO and (h) S@NH_3_-rGO samples; EDX analysis of the S@rGO sample: (b) carbon and (c) sulfur; S@en-GO sample: (e) carbon, (f) sulfur and (g) nitrogen: and S@NH_3_-rGO sample: (i) carbon, (j) sulfur and (k) nitrogen. Figure S4. (a) Cycle voltammograms at 0.05, 0.1, 0.2, 0.4 and 0.6 mV s^−1^ between 3.0 and 1.5 V for the S@rGO electrode; (b) Impedance spectra for S@rGO electrode; (c) Graphical representation of peak intensity versus sweep speed elevated to a half for the S@rGO electrode; and (d) Graphical representation of real impedance component (Z_real_) versus ω^−1/2^ in the Warburg region to evaluate the σ term for Equation (2) for the S@en-GO, S@NH_3_-rGO and S@rGO electrodes. Figure S5. (a) Long-term discharge/charge capacity values as a function of the cycle number and the coulombic efficiency at C/10 rate for the S@rGO electrode; (b) Long-term discharge/charge capacity values as a function of the cycle number and the coulombic efficiency at C/5 rate for S@en-rGO and S@rGO electrodes. Figure S6. Charge/discharge profiles at C/10, C/5, C/2, 1C, 2C, 5C, 10C, 12C, and 15C rates of the Li-S cells with theS@NH_3_-rGO electrode. Figure S7. SEM images of the NH_3_-rGO electrode (a) before and (b) after rate capability test (C/10, C/8, C/5, C/3, C/2, 1C and 2C; 5 cycles each and C/10 again during 50 more cycles). Table S1. Values of the impedances’ components, *χ*^2^, τ and Li^+^ ion diffusion coefficients (cm^2^ s^−1^) of the S@en-rGO, S@NH_3_-rGO and S@rGO electrodes obtained by applying the Equation (2). Table S2. Li^+^ ion diffusion coefficients (cm^2^ s^−1^) of the S@en-rGO, S@NH_3_-rGO and S@rGO electrodes obtained by applying the Randles-Sevcik Equation (1).
