# Supplementary material for: Effect of Nitrogen Dopant Agents in the Performance of Graphene-Based Cathodes for Li-S Batteries
Source: Nanomaterials (Basel). 2024 Mar 8;14(6):489. doi: 10.3390/nano14060489 (PMC10974078; doi:10.3390/nano14060489)
Supplement: Supplementary file 1 [file nanomaterials-14-00489-s001.zip › nanomaterials-2889968-supplementary.pdf]

Supplementary Material

Effect of Nitrogen Dopant Agents in the Performance of Graphene-Based Cathodes for Li-S Batteries

Adrián Licari, Almudena Benítez *, Juan Luis Gómez-Cámer, Rafael Trócoli and Álvaro Caballero *

Dpto. Química Inorgánica e Ingeniería Química, Instituto Químico para la Energía y el Medioambiente (IQUEMA), Facultad de Ciencias, Universidad de Córdoba, 14071, Córdoba, Spain.

* Correspondence: q62betoa@uco.es (A.B.); alvaro.caballero@uco.es (Á.C.); Tel.: +34-957218621 (A.B.); +34-957218620 (Á.C.)

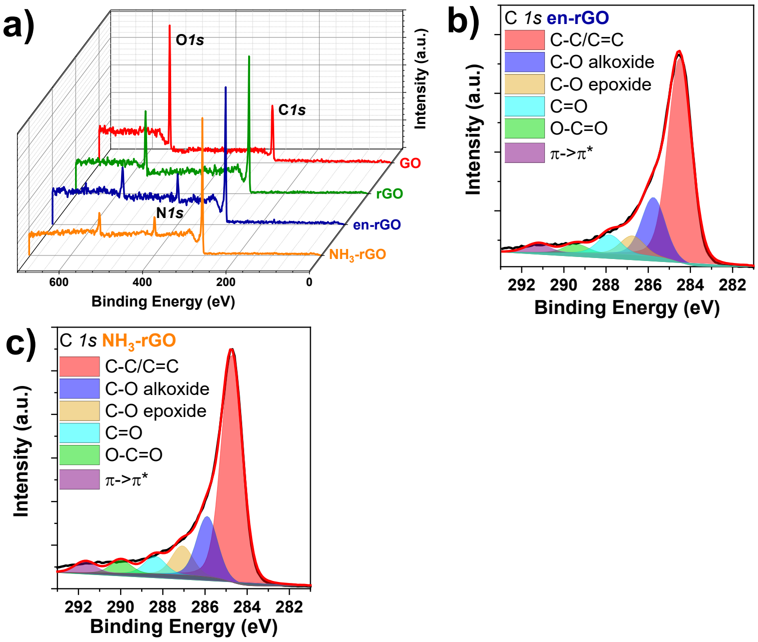

Figure S1. a) XPS survey for the GO, rGO, en-rGO and NH_3-rGO samples; XPS spectrum of the C*1s* signal for the b) en-GO, and c) NH_3-rGO samples.

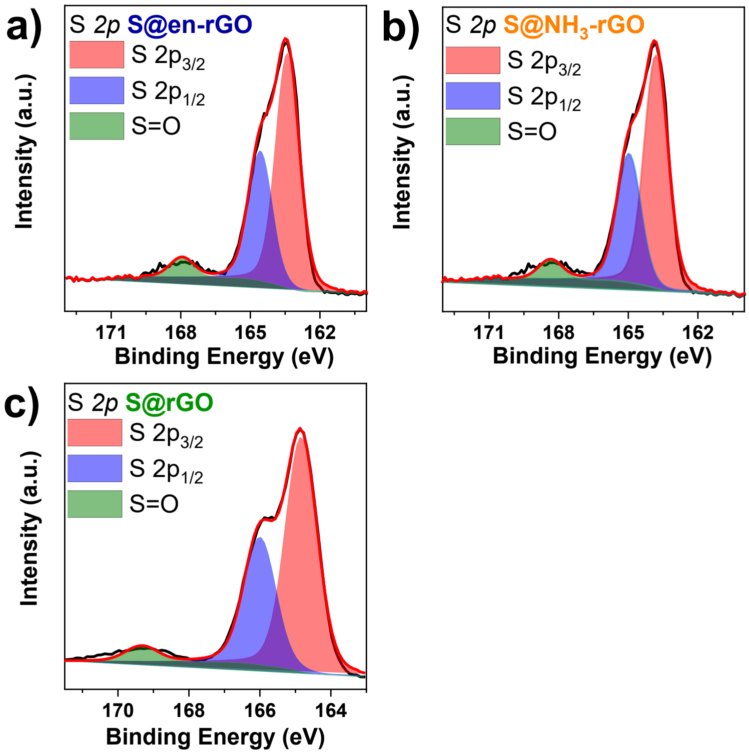

Figure S2. XPS spectrum of the S *2p* signal for the a) S@en-GO, b) S@NH_3-rGO, and c) S@rGO samples.

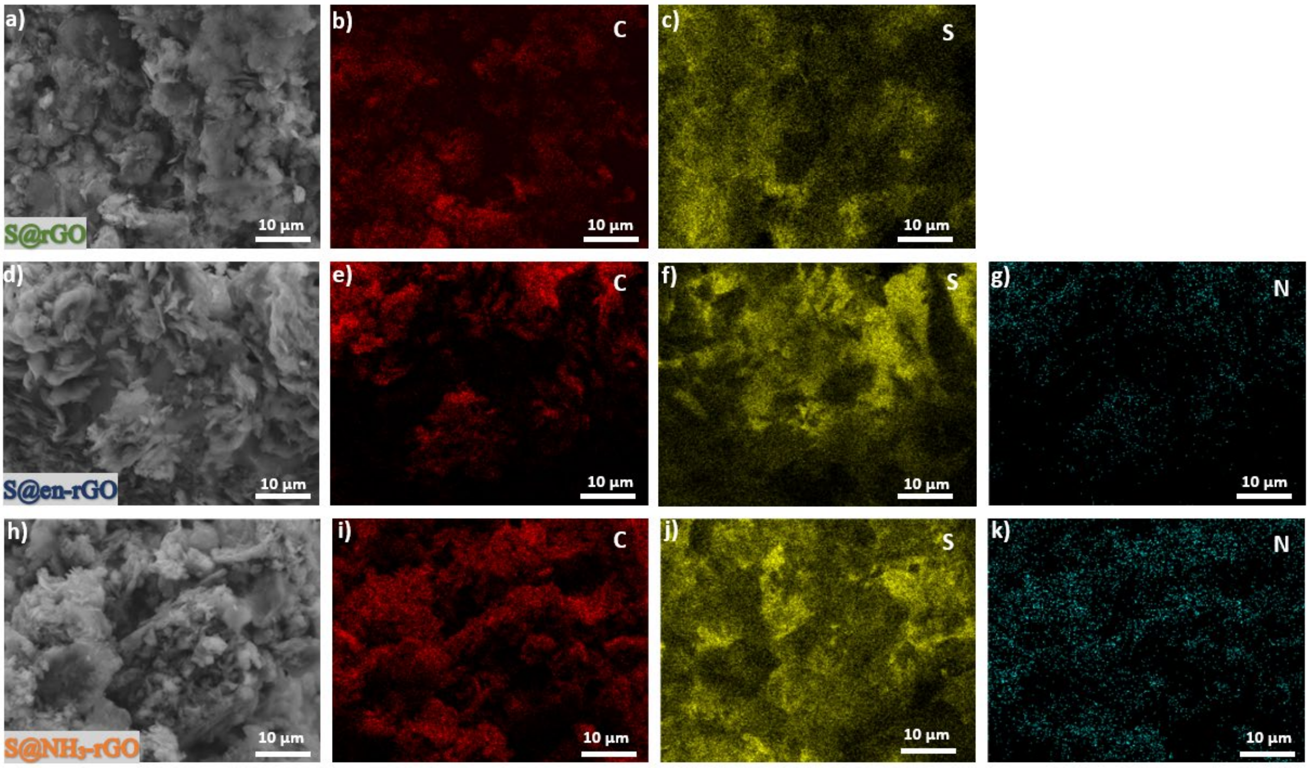

Figure S3. SEM images of the a) S@rGO, d) S@en-GO and h) S@NH_3-rGO samples; EDX analysis of the S@rGO sample: b) carbon and c) sulfur; S@en-GO sample: e) carbon, f) sulfur and g) nitrogen: and S@NH_3-rGO sample: i) carbon, j) sulfur and k) nitrogen.

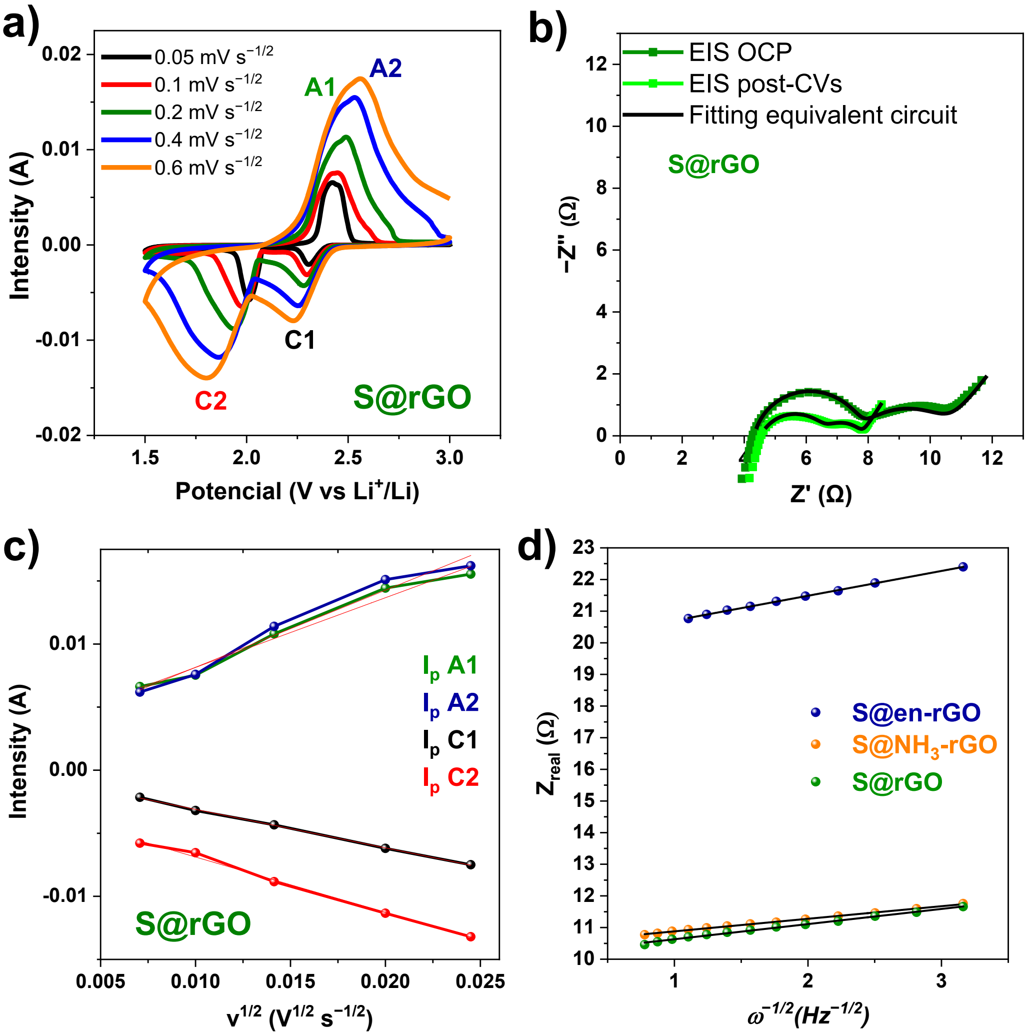

Figure S4. a) Cycle voltammograms at 0.05, 0.1, 0.2, 0.4 and 0.6 mV s^{-1} between 3.0 and 1.5 V for the S@rGO electrode; b) Impedance spectra for S@rGO electrode; c) Graphical representation of peak intensity versus sweep speed elevated to a half for the S@rGO electrode; and d) Graphical representation of real impedance component (Z_{real}) versus $\omega^{-1/2}$ in the Warburg region to evaluate the σ term for equation (2) for the S@en-GO, S@NH_3-rGO and S@rGO electrodes.

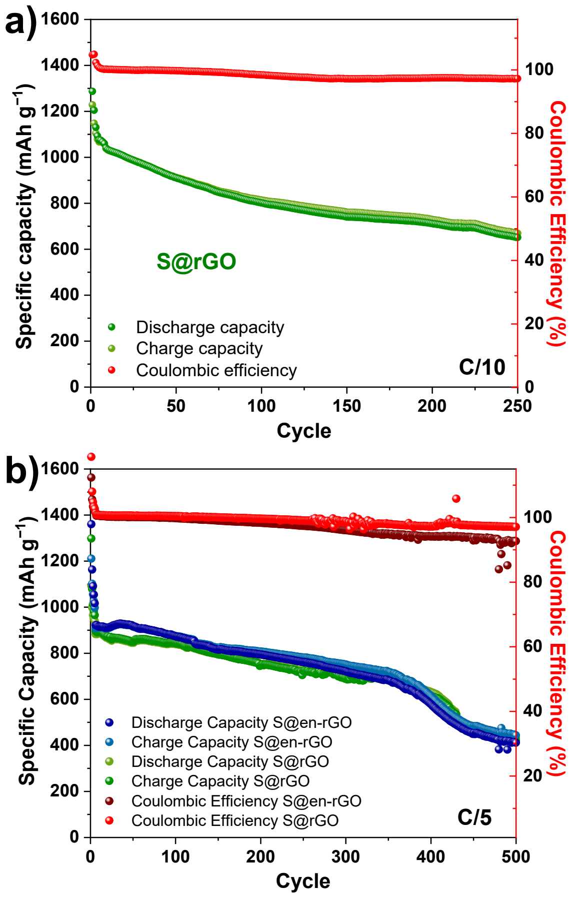

Figure S5. a) Long-term discharge/charge capacity values as a function of the cycle number and the coulombic efficiency at C/10 rate for the S@rGO electrode; b) Long-term discharge/charge capacity values as a function of the cycle number and the coulombic efficiency at C/5 rate for S@en-rGO and S@rGO electrodes.

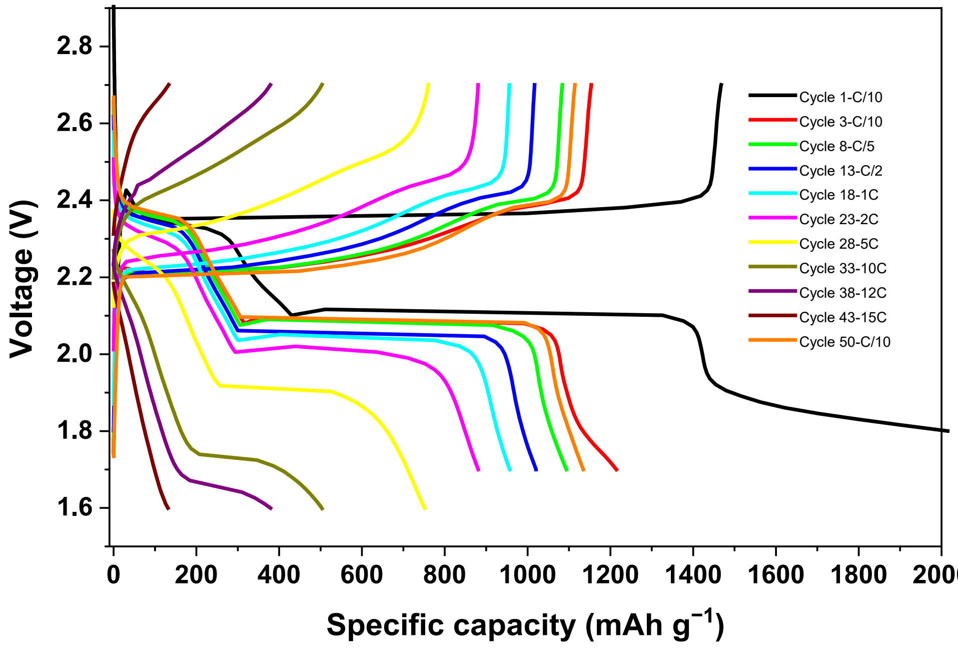

Figure S6. Charge/discharge profiles at C/10, C/5, C/2, 1C, 2C, 5C, 10C, 12C, and 15C rates of the Li-S cells with theS@NH3-rGO electrode.

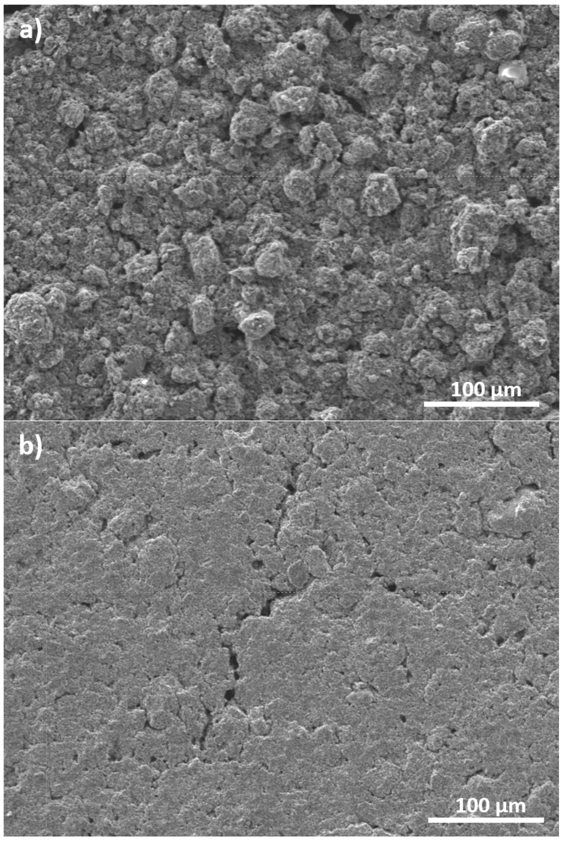

Figure S7. SEM images of the NH_3-rGO electrode (a) before and (b) after rate capability test (C/10, C/8, C/5, C/3, C/2, 1C and 2C; 5 cycles each and C/10 again during 50 more cycles).

Table S1. Values of the impedances' components, χ^2, τ and Li^+ ion diffusion coefficients ($cm^2\ s^{-1}$) of the S@en-rGO, S@NH_3-rGO and S@rGO electrodes obtained by applying the equation (2).

Samples	EIS	χ^2	R_e (Ω)	R_{int} (Ω)	R_{ct} (Ω)	τ_{ct} (s)	σ ($\Omega\cdot s^{-1/2}$)	D_{EIS}
**S@en-rGO**	OCP	$2.80\cdot10^{-4}$	5.42	11.49	3.86	0.014	0.78	$2.05\cdot10^{-9}$
	Post-CV	$4.90\cdot10^{-4}$	4.65	2.41	3.57			
**S@NH_3-rGO**	OCP	$1.20\cdot10^{-3}$	4.51	3.52	2.94	0.004	0.40	$7.96\cdot10^{-9}$
	Post-CV	$1.48\cdot10^{-3}$	3.48	2.36	0.94			
**S@rGO**	OCP	$1.10\cdot10^{-3}$	4.33	3.40	2.97	0.008	0.48	$5.49\cdot10^{-9}$
	Post-CV	$1.27\cdot10^{-3}$	4.54	2.08	1.20			

Table S2. Li^+ ion diffusion coefficients ($cm^2\ s^{-1}$) of the S@en-rGO, S@NH_3-rGO and S@rGO electrodes obtained by applying the Randles-Sevcik equation (1).

	**S@en-rGO**	**S@NH_3-rGO**	**S@rGO**
**A1**	0.60	0.62	0.55
**A2**	0.64	0.68	0.61
**C1**	-0.29	-0.34	-0.31
**C2**	-0.40	-0.50	-0.44
**D_{Li} (A1)**	$2.89\cdot10^{-6}$	$3.10\cdot10^{-6}$	$2.43\cdot10^{-6}$
**D_{Li} (A2)**	$3.27\cdot10^{-6}$	$3.69\cdot10^{-6}$	$2.97\cdot10^{-6}$
**D_{Li} (C1)**	$6.59\cdot10^{-7}$	$9.34\cdot10^{-7}$	$7.40\cdot10^{-7}$
**D_{Li} (C2)**	$1.29\cdot10^{-6}$	$2.02\cdot10^{-6}$	$1.53\cdot10^{-6}$
